# Biogeography of Deep-Sea Benthic Bacteria at Regional Scale (LTER HAUSGARTEN, Fram Strait, Arctic)

**DOI:** 10.1371/journal.pone.0072779

**Published:** 2013-09-02

**Authors:** Marianne Jacob, Thomas Soltwedel, Antje Boetius, Alban Ramette

**Affiliations:** 1 HGF-MPG Research Group for Deep-Sea Ecology and Technology, Alfred-Wegener-Institut Helmholtz-Zentrum für Polar- und Meeresforschung, Bremerhaven, Germany; 2 Max Planck Institute for Marine Microbiology, Bremen, Germany; Argonne National Laboratory, United States of America

## Abstract

Knowledge on spatial scales of the distribution of deep-sea life is still sparse, but highly relevant to the understanding of dispersal, habitat ranges and ecological processes. We examined regional spatial distribution patterns of the benthic bacterial community and covarying environmental parameters such as water depth, biomass and energy availability at the Arctic Long-Term Ecological Research (LTER) site HAUSGARTEN (Eastern Fram Strait). Samples from 13 stations were retrieved from a bathymetric (1,284–3,535 m water depth, 54 km in length) and a latitudinal transect (∼ 2,500 m water depth; 123 km in length). 454 massively parallel tag sequencing (MPTS) and automated ribosomal intergenic spacer analysis (ARISA) were combined to describe both abundant and rare types shaping the bacterial community. This spatial sampling scheme allowed detection of up to 99% of the estimated richness on phylum and class levels. At the resolution of operational taxonomic units (97% sequence identity; OTU_3%_) only 36% of the Chao1 estimated richness was recovered, indicating a high diversity, mostly due to rare types (62% of all OTU_3%_). Accordingly, a high turnover of the bacterial community was also observed between any two sampling stations (average replacement of 79% of OTU_3%_), yet no direct correlation with spatial distance was observed within the region. Bacterial community composition and structure differed significantly with increasing water depth along the bathymetric transect. The relative sequence abundance of Verrucomicrobia and Planctomycetes decreased significantly with water depth, and that of Deferribacteres increased. Energy availability, estimated from phytodetrital pigment concentrations in the sediments, partly explained the variation in community structure. Overall, this study indicates a high proportion of unique bacterial types on relatively small spatial scales (tens of kilometers), and supports the sampling design of the LTER site HAUSGARTEN to study bacterial community shifts in this rapidly changing area of the world’s oceans.

## Introduction

Biogeographic patterns have been identified at global and regional scales for marine microbes, (e.g., [1], [2]). In most studies, these patterns may be explained by a combination of spatial distance effects and contemporary environmental variations in physical, chemical and biological factors [3]. In an environmentally relatively uniform habitat such as the deep-sea floor, the influence of horizontal geographical distance on community patterns is likely related to dispersal limitation, resulting in a distance-decay relationship [2], [4]. In a completely uniform habitat, this relationship could be entirely caused by drift [5]. In naturally patchy environments, selection pressures and historical processes will also play an important role [6]. However, so far it remains unclear at what spatial scales these different processes act on bacterial communities in deep-sea sediments. Information on such spatial patterns is not only important to understand the distribution range of bacterial species, it is also a prerequisite for monitoring and evaluating temporal variations in deep-sea ecosystems, for example by climate change and other anthropogenic disturbances [7], or for the implementation of marine protected areas [8].

A strong impact of spatial distance together with water depth and surface water productivity on variation in marine benthic bacterial community structure has already been detected on a global scale in coastal and deep-sea sediments [2]. In the South Atlantic, correlations between spatial distances and bacterial community structures at intermediate scale (up to 1,200 km distance), large scale (up to 3,500 km distance) and basin wide scale (up to 18,000 km distance) were observed [1]. Also in the Arctic sector, geographically related patterns of bacterial diversity were suggested based on surface sediment samples from two shallow (40 and 447 m water depth) and two deep stations (3,000 and 3,850 m water depth) in the Chukchi Sea and Canada Basin [9], while no such patterns were found in the western Greenland Sea (2,747–3,395 m water depth; 16 stations) [10]. Along the Siberian continental margin an energy-diversity relationship was found, which was tightly coupled to water depth differences, while accounting for spatial factors (37–3,427 m water depth; 17 stations) [11].

**Figure 1:**
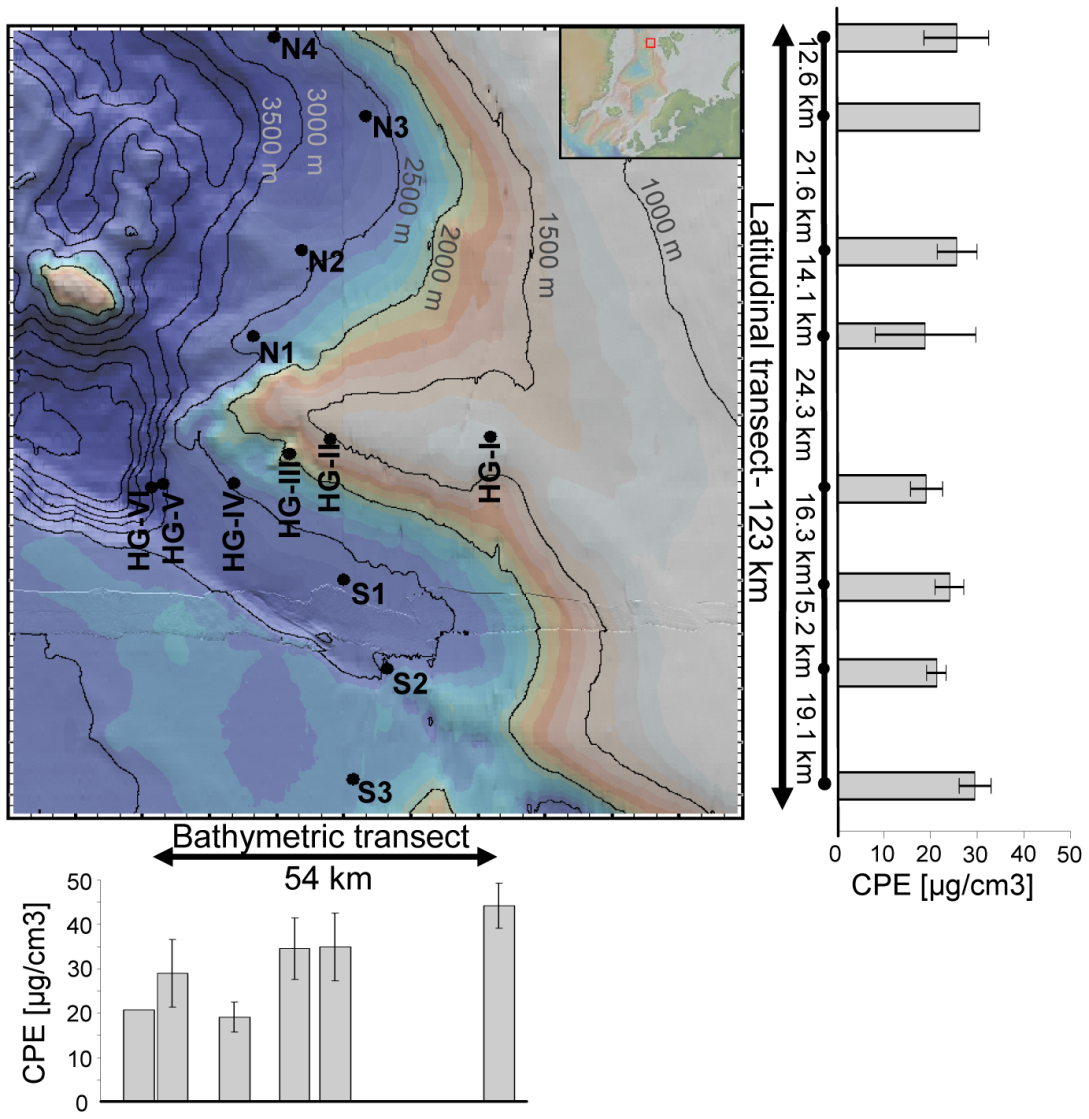
Location of sampling stations of the LTER site HAUSGARTEN and corresponding pigment concentrations (CPE). Distances in km between sampling stations were calculated from latitude or longitude only for the latitudinal and bathymetric transect, respectively. Map created with GeoMapApp [70].

In this study of the Arctic Long-Term Ecological Research (LTER) site HAUSGARTEN in Fram Strait [12], we investigated the impact of spatial distance, water depth and environmental parameters related to food availability (phytodetrital pigments) and biomass on bacterial diversity and community structure, on a local to regional scale (∼ 1–100 km distances). The part of the LTER site studied here covered 13 sampling sites arranged along two perpendicular transects. A bathymetric transect that spans water depths of 1,284 to 3,535 m (54 km length) and thereby incorporating a difference in phytodetritus input, and also a latitudinal transect covering a distance of 123 km along similar water depths (∼ 2,500 m), lacking such a strong gradient in food availability [13] (Figure 1). This allowed testing the hypotheses a) that spatial distances of 10–100 km can structure bacterial communities of the deep-sea floor; and b) that spatial patterns of bacterial communities can be linked to variations in food availability caused by different fluxes of particulate organic matter at different water depths. The objectives of this study were accordingly 1) to describe changes in bacterial diversity at the regional scale both in terms of local richness and community turnover, 2) to determine whether specific spatial and environmental factors explain changes in diversity patterns, and 3) to identify bacterial types that may be specifically affected by spatial or environmental factors.

## Materials and Methods

### Study Site

Fram Strait is the only deep-water connection to the Arctic Ocean. Here, warm Atlantic water masses enter the Arctic Ocean through the West Spitsbergen current, while cold Polar waters exit through the East Greenland Current [14], [15]. Over the last decade, significant changes in sea ice distribution, temperature fluctuations of Atlantic water masses [16], changes in the biological composition of the water column [17], [18] and the composition of export fluxes [19] have been observed. Due to a high efficiency of benthic-pelagic coupling [20], [21], [22], the ongoing changes of Arctic surface ocean conditions are predicted to directly affect the benthic environment [23], [24], which depends on organic matter input from the more productive zone of the upper water column [25]. Main contributors to benthic carbon processing in Fram Strait are bacteria [26], which make up the major fraction of the small benthic infaunal biomass (up to 95%) [27]. Previous investigations on the bacterial community structure of this region include *in situ* experiments of bacterial colonization of artificial and deep-sea sediments [28], bacterial community response to chitin enriched sediments over different time scales [29] and around biogenic structures [30]. Natural spatial variation in benthic bacterial diversity was also investigated along a canyon at the Greenland continental rise over a distance of 200 km [10].

### Sampling Strategy

During the cruise ARK-XXIV/2 in July 2009 with the German research ice-breaker RV Polarstern to the LTER site HAUSGARTEN [12] west of Spitsbergen (Figure 1), samples of virtually undisturbed sediments where taken using a TV-guided multiple corer (TV-MUC) at 78.6–9.7°N and 3.5–6°E (Table S1). Six stations (HG-I to HG-VI) along a bathymetric transect from East to West from 1,284 m down to 3,535 m water depth as well as a latitudinal transect with eight stations (N1 to N4, HG-IV, and S1 to S3) at about 2,500 m water depth were sampled (Table S1). The most northern stations (N3 and N4) as well as the deepest station sampled in this study (HG-VI) were partly ice covered during sampling. TV-MUC cores were sub-sampled using modified 10-ml syringes (2 cm in diameter), sub-divided into 1-cm layers and only the uppermost centimeter representing the most active community was analyzed in this study [31]. Necessary permits for sampling were obtained from the Norwegian authorities (Fisheries directorate). The locations sampled are not privately-owned or protected areas, and the field studies did not involve endangered or protected species.

### Biotic and Abiotic Factors

Sample processing for all environmental parameters was done as described in [22]. In brief, concentrations of chlorophyll *a* and its degradation products phaeopigments, here summarized as chloroplastic pigment equivalents (CPE) [32], were determined using a Turner fluorometer. CPE concentrations serve as an indicator for food availability in form of phytodetritus originating from photosynthetic production in surface ocean layers. Porosity of sediments was assessed by the weight loss of wet sediment samples dried at 60°C. Phospholipids, indicating the total microbial biomass, were analysed by gas chromatography, and particulate proteins, indicating the biomass of detrital matter, were analysed photometrically [33]. Data is available at doi.pangaea.de/10.1594/PANGAEA.744673 -doi.pangaea.de/10.1594/PANGAEA.744685 (Table S1).

### DNA Extraction and Purification

Sediment from the uppermost centimeter originating from three different TV-MUC cores was pooled. Total DNA was extracted from 1 g of this homogenized slurry (comprising on average 4.22×10^8^ bacterial cells as determined by acridine orange direct counting [34]) using the UltraClean Soil DNA Isolation Kit (MO BIO Laboratories, Inc., Carlsbad, CA, USA) according to the manufacturer’s instructions for maximum yields. Elution was carried out using 4×50 µl Tris-EDTA buffer (Promega, Madison, WI, USA). DNA extracts that showed a final DNA concentration lower than 4 ng µl^−1^ (determined spectrophotometrically using a NanoDrop Spectrophotometer ND 1000, Thermo Fisher Scientific Inc., Wilmington, DE, USA) were purified via isopropanol precipitation. Final DNA concentrations ranged from 4–12 ng µl^−1^.

### Automated Ribosomal Intergenic Spacer Analysis (ARISA)

ARISA PCR consisted of 1×Eppendorf PCR buffer (5′Prime Inc., Gaithersburg, MD, USA), 0.25 mM desoxynucleoside-triphosphate mix (Promega), 0.3 g l^−1^ bovine serum albumin, 0.4 µM of each primer, 0.05 units Eppendorf Taq (5′Prime Inc.) and 20–25 ng DNA (determined spectrophotometrically using a Tecan Infinite 200, Tecan Group Ltd., Switzerland) in a total volume of 50 µl. Primers were used and PCR amplification (in triplicates per sample), separation of fragments by capillary electrophoresis, evaluation of signals and binning into operational taxonomic units (OTU) was done as described previously [35]. In order to get reliable data for statistical analyses, only those OTU that occurred in at least two of the PCR triplicates were kept for further analyses and their relative peak areas were averaged to produce one complete fingerprint per sample.

### 454 Massively Parallel Tag Sequencing (MPTS)

Extracted DNA was amplified at the Marine Biological Laboratory (Woods Hole, MA, USA) according to the protocol published on http://vamps.mbl.edu, using primers targeting the V4–V6 region of the bacterial 16 S rRNA gene. SFF files were deposited in the GenBank Sequence Read Archives (www.ncbi.nlm.nih.gov) under BioProject ID: PRJNA208712. Preparation of flowgrams and transformation into an OTU- by- Sample table were conducted with “mothur” [36] according to the standard operating procedure (SOP [37]) including the implemented denoising algorithm. Alignment of denoised sequences and taxonomic affiliation were carried out using the SILVA reference file for bacteria [38] (downloaded from http://www.mothur.org in March 2012) and chimeric sequences were identified using the mothur implemented *uchime* program. Cleaned sequences were clustered at a 97% identity level into operational taxonomic units (OTU_3%_) and the dataset was normalized by the total amount of sequences per sample to get relative abundances. To investigate the rare biosphere [39] we considered: a) OTU_3%_ that occurred with only one sequence in the whole denoised dataset (absolute singletons), called SSO_abs_ and b) OTU_3%_ that consisted of only one sequence in at least one sample, and were not absolute singletons (relative singletons or SSO_rel_), so the total number of sequences for any SSO_rel_ was larger than one [40]. Taxonomic assignment up to the genus level was possible for 40% of all OTU_3%,_ but only 4% of all OTU_3%_ were assigned up to the species level. Therefore we only considered annotation up to genus level for subsequent analyses.

### Statistical Analyses

Chao1 richness estimates per sample were calculated on a normalized subset based on the sample with lowest number of OTU_3%_ (i.e. HG-II, 3,716 OTU_3%_). Turnover of OTU was calculated as percentage of pairwise shared, lost or gained OTU relative to the total number of OTU in the two samples. Shared OTU are those appearing in both samples, lost OTU are only present in the first sample and gained OTU are only present in the second sample. To compare bacterial classes found in this study to those found in other studies (i.e. [2], [11]), we only considered the shared classes and then calculated their mean relative sequence abundances for each subset. To determine whether class proportions obtained in this study could be predicted from the previous studies, we used linear regression and determined whether the slope coefficients were significantly different from one by calculating the 95% confidence intervals of the respective slope coefficients (e.g. [35]).

Dissimilarity matrices based on community data and environmental tables were calculated using Bray-Curtis and Euclidean distances, respectively. Homogeneity of group dispersions were determined by calculating the average distance of a group member to the median of the group [41] and the central station HG-IV was included in both transects. Non-metric multidimensional scaling (NMDS) was performed together with a minimum-spanning tree between samples connecting nearest neighbours (i.e. the most similar stations) in terms of similarity of their community structure to visualize pairwise community similarities. Mantel tests with 999 Monte-Carlo permutations were used to test for the significance of Spearman correlations between dissimilarity matrices or dissimilarity matrices and environmental parameters.

Except for longitude, latitude, spatial distance and water depth, all parameters were normalized by log_10_ transformation to meet the assumptions for regression analysis (see [42]). Distances between sampling stations were calculated in kilometer from only longitude or latitude for the bathymetric and latitudinal transect, respectively. Spatial distance between sampling stations of all stations were calculated with both, longitude and latitude. Redundancy analyses (RDA) were used to explore the degree of variation in community datasets that can be explained by environmental parameters. In order to look for pure effects of certain environmental parameters, canonical variation partitioning [42] was performed using the forward selected contextual parameters water depth and CPE concentrations. We used CPE concentrations as they explained more of the variability than chlorophyll *a* or phaeopigments alone. When referring to behaviour of certain taxa, the OTU_3%_ data was pooled using the “taxa.pooler.1.2” of the MultiCoLA software package [43] which groups all OTU_3%_ that were assigned to a taxonomic group at a predefined taxonomic level. OTU_3%_ that were not classified at a certain taxonomic level were combined into one group. All analyses were performed in R (v.2.14.1) [44] using *vegan* [45], *permute* [46] and *MASS* [47] packages.

## Results and Discussion

Biogeographic patterns of surface sediment bacterial communities were investigated at the Arctic LTER site HAUSGARTEN (∼79°N, 4°E; Figure 1, Table S1). Shifts in bacterial community structure were investigated using automated ribosomal intergenic spacer analysis (ARISA) and 454 massively parallel tag sequencing (MPTS) of the V4–V6 variable regions. We found consistent community patterns derived from both data types at different taxonomic resolution levels (Table S2), thus we mostly focused on results based on MPTS data, including some comparisons to the patterns detected by ARISA.

### Richness of Bacterial Types

Using MPTS data, a total of 41 phyla, 78 classes, 136 orders, 215 families, and 410 genera were identified (Table S3). Most of the OTU_3%_ belonged to the phylum Proteobacteria (47% of all OTU_3%_) with the most abundant classes being Gammaproteobacteria (23%), Deltaproteobacteria (15%) and Alphaproteobacteria (7%). The second most OTU_3%_ abundant phylum was Bacteroidetes (9%) with, among others, the classes Flavobacteria (3%) and Sphingobacteria (5%). Other abundant phyla were Actinobacteria (3%), Acidobacteria (5%), and Verrucomicrobia (4%). Those proportions barely changed when excluding SSO_abs_ from the dataset. These phyla and classes were also found as abundant members of Arctic sediments from the Pacific sector [9], in a fjord off Svalbard [48], the Siberian continental margin [11], as well as in other benthic environments [2].

**Figure 2:**
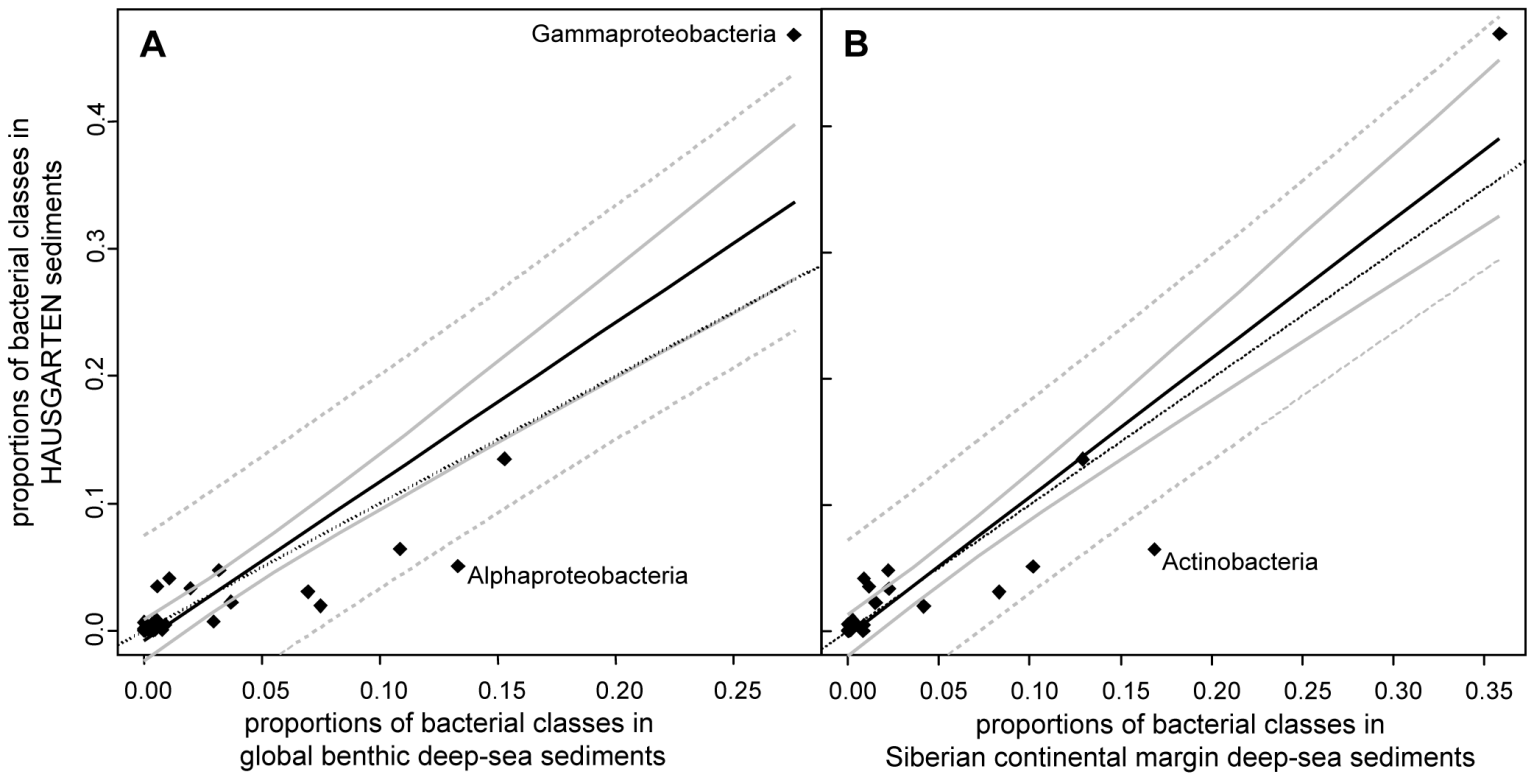
Comparison of bacterial classes in sediments from HAUSGARTEN with other datasets. A: Globally distributed sediments; B: sediments from the Siberian continental margin. The solid lines indicate the best fit using linear regression; solid grey lines indicate 95% confidence intervals; dotted grey lines indicate predicted intervals at a 95% confidence level; dotted black lines indicate the case where equal proportions were found in the datasets being compared (y = x).

The mean proportions of bacterial classes inhabiting HAUSGARTEN sediments were in very good agreement (R^2^ = 0.78, p<0.001; determined by linear regression; Figure 2) with those predicted for globally distributed benthic deep-sea samples (262–5,347 m water depth), indicating a typical deep-sea microbiome [2]. Differences from the global average included for example lower Alphaproteobacteria and higher Gammaproteobacteria relative sequence abundances at HAUSGARTEN. When considering Siberian continental margin sediments (534–3,427 m water depth) [11], we found an even better relationship for mean class proportions (R^2^ = 0.85, p<0.001; Figure 2). Those observations were corroborated by determining the slope coefficients of each comparison, and slope coefficients of 1.25±0.24 (95% confidence interval assuming a Student’s *t* distribution with 30 degrees of freedom) and 1.1±0.19 (24 degrees of freedom), were obtained for the comparison with the global dataset and the Siberian margin dataset, respectively. This shows that the best model (i.e. a slope coefficient of 1 and higher explained variance) is obtained in the latter case when only considering sediments from the Arctic.

Chao1 richness estimates were on average 3,010±642 OTU_3%_ per sample at each station (Table S4), which is comparable to sediments from the Siberian continental margin [11] and higher than for samples from the deep Arctic Ocean water column [49]. Interestingly the variation in richness (coefficient of variation 0.21) was close to that observed for biomass (phospholipid concentration per sample, CV = 0.25 based on 12±3 nmol ml^−1^; Table S1). We found no correlation of the number of OTU_ARISA_, nor of observed or estimated richness of OTU_3%_ per sample with pigment concentrations (CPE), water depth (Table S5) or with any other contextual parameter (latitude, porosity, particulate protein concentrations, phospholipid concentrations; data not shown). These observations did not change when removing singletons from the dataset (data not shown). Our findings differ from a previous investigation of the oligotrophic Siberian continental margin where both, numbers of OTU_ARISA_ and estimated richness of OTU_3%_, correlated positively with phaeopigment concentrations below 4 µg cm^−3^ [11]. However, in Fram Strait, phaeopigment concentrations were considerably higher (13–37 µg cm^−3^) than at the Siberian continental margin (<8 µg cm^−3^) [11]. This may indicate that, within the range of phytodetritus supply to the deep Fram Strait (Table S1), the observed local variations in bacterial richness might be driven by other factors than energy supply and water depth. For example, it is possible that the locally differing assemblages of benthic fauna [13], [50], [51] have an impact on local patterns in bacterial richness for example, by altering the sediment-water interface and particle deposition or grazing (see [30], [52], [53]), which remains to be further investigated.

### Sampling Effect on Diversity Discovery

The increase of newly detected OTU_3%_ with every sampled station was linear (Figure S1B). By sampling 12 of 13 stations, 95% of observed OTU_3%_ were detected and 36% of estimated richness was recovered, when considering all stations (Table S3). The OTU_3%_ accumulation curve could not reach a plateau because of the high numbers of singletons in the dataset (62% of all OTU_3%_). In contrast, the OTU accumulation curve for ARISA data did reach a plateau and only nine stations were needed to recover 95% of all observed OTU_ARISA_ (Figure S1A). This reflects the technical limitations of ARISA such as the maximum number of detectable OTU_ARISA_ (here 450) and 16–23 S length identity between different genera or species [54] (see Text S1).

**Figure 3:**
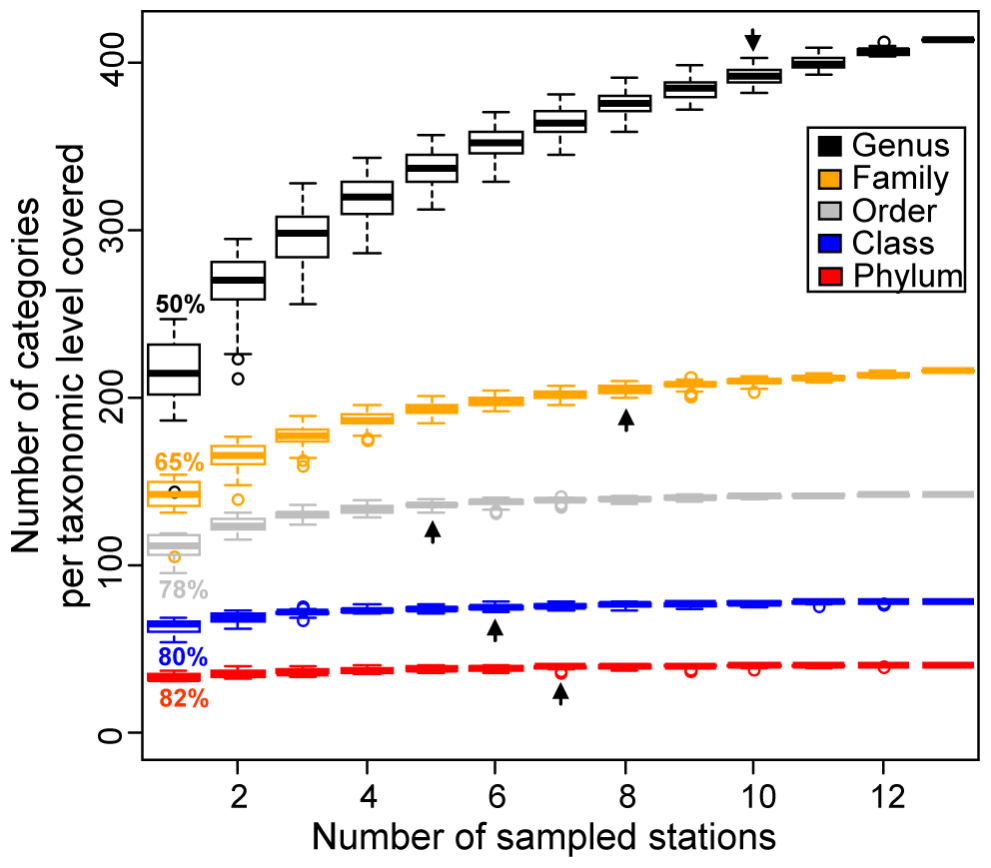
Accumulation curves per taxonomic category based on MPTS data. Arrows indicate how many stations are needed to recover 95% of categories per taxonomic level. The percentages indicated for n = 1 station correspond to how much diversity would be recovered on average by randomly sampling only one station.

To investigate the effects of taxonomic resolution, we used the taxonomic information associated to each OTU_3%_ from phylum to genus, according to [43] (see Table S3). Only 1.36% of all OTU_3%_ could not be assigned to a known phylum. Taking only seven stations into account, at least 95% of all observed phyla, classes or orders were recovered; in contrast, sampling of ten stations was needed to recover 95% of all occurring genera in the dataset (Figure 3). Considering all stations, 99% of the estimated richness of phyla and classes were described and 77% of the estimated richness of genera (Table S3). In order to determine which transect added most to the total diversity – the bathymetric transect covering water depth together with food availability differences and spatial distance, or the latitudinal transect representing mostly pure spatial distance - we analysed both transects separately, but compared the recovered diversity with that of the whole dataset. From the latitudinal transect alone 5, 6, 5 and 8 stations were needed to cover 95% of all observed phyla, classes, orders and families, respectively, in the entire HAUSGARTEN dataset. With all stations from the latitudinal transect, 99% of the estimated total richness at the phylum, class and order level were recovered, 95 and 92% at the family and genus level, respectively. At the OTU_3%_ level, 78% of observed and 28% of estimated total richness was recovered. Along the bathymetric transect, 89%, 93%, 93%, 75% and 81% of the estimated total richness was recovered at the phylum, class, order, family and genus level, respectively. Only 50% of all observed OTU_3%_ were found at stations from the bathymetric transect, and only 18% of estimated richness could be recovered by sampling the six stations along this transect. Hence, a high amount of bacterial diversity came from the latitudinal transect. By sampling only this transect, most of the diversity discovery at coarse taxonomic levels was covered. The latitudinal transect hosted four unique candidate divisions WS1, OP9, SR1 and WCHB1–60, which did not occur in samples from the bathymetric transect. Overall, the near-complete coverage of diversity at coarse taxonomic resolution shows that our sampling scheme was suitable to examine bacterial diversity at the regional scale. Still, with every additional sample, new families, genera and, most of all, OTU_3%_ could be detected.

### Community Turnover and Structure along the Two Transects

On average 21±2% OTU_3%_ (32±3% when removing SSO_abs_) were shared between any two samples at HAUSGARTEN (Table S6) which is higher than shared OTU_3%_ between coastal and deep-sea surface sediments (∼ 14 OTU_3%_) around the whole globe [2]. Overall, no correlation of community composition (similarities in the presence and absence of OTU_3%_) with spatial distance between any two samples was observed (p = 0.557), neither for the whole data set, nor for samples of the latitudinal transect (13–123 km difference; p = 0.246) or of the bathymetric transect alone (2–52 km difference; p = 0.107) when based on MPTS data including singletons. Removing absolute singletons from the dataset led to the same conclusions (data not shown). In contrast, community composition of samples from the bathymetric transect based on ARISA – known to detect the more abundant types - significantly correlated with spatial distance (r = 0.83, p = 0.013).

**Figure 4:**
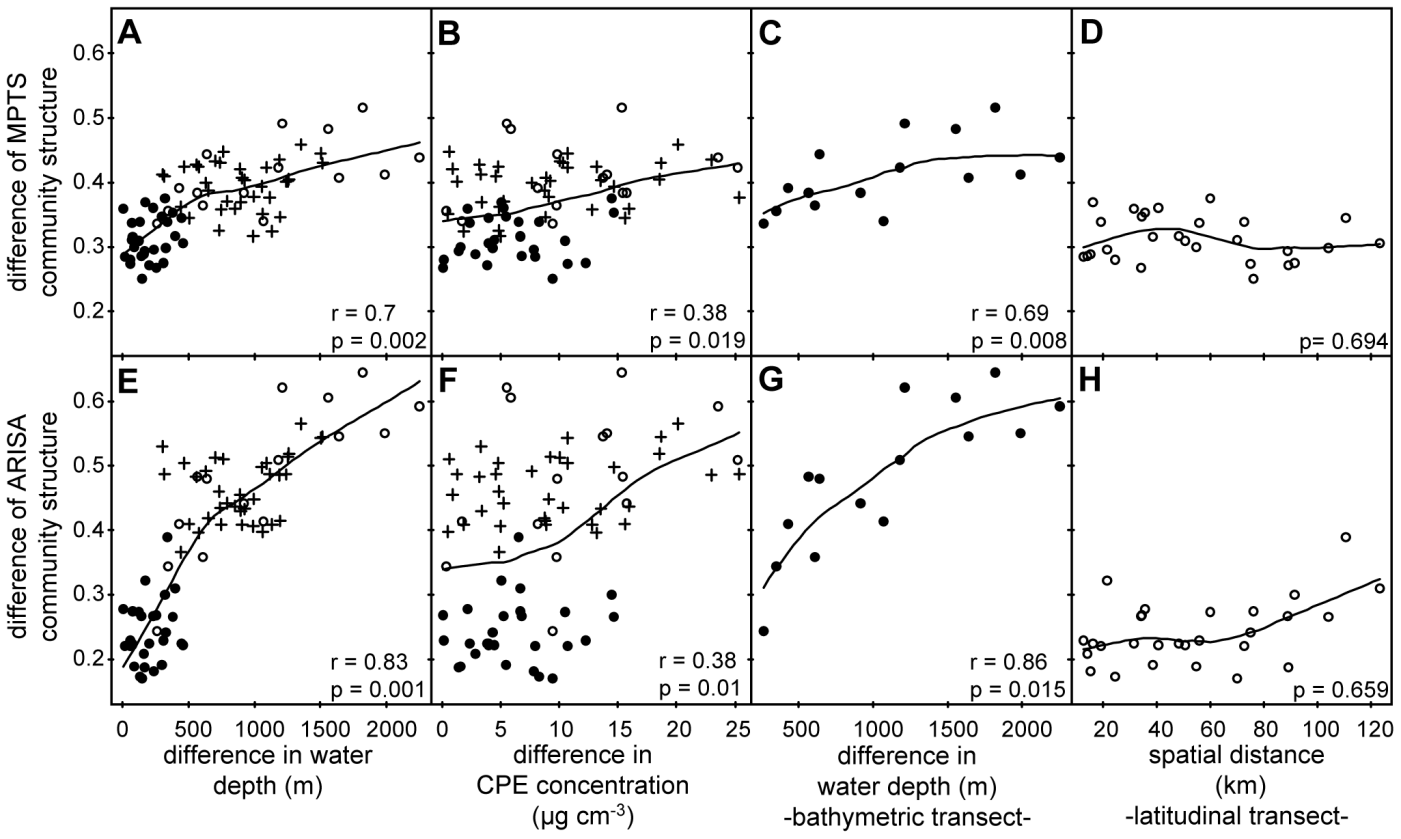
Changes in bacterial community structure with water depth and CPE concentrations and along spatial distance for the two transects. The plots A, B, C and D are based on MPTS data, plots in E, F, G and H are based on ARISA data. Filled circles indicate comparisons of samples from the latitudinal transect, open circles indicate comparisons of samples from the bathymetric transect, crosses indicate comparisons across transects. C, D, G and H are based on a subset of 6 and 8 samples for the bathymetric and latitudinal transects, respectively. Mantel tests were used to assess the significance of Spearman’s correlation coefficients (r) based on 1000 permutations.

Dissimilarities in community composition significantly correlated with water depth differences along the bathymetric transect (r = 0.56, p = 0.032; r = 0.62, p = 0.034 when removing SSO_abs_; 263–2,251 m water depth differences). Pairwise shared OTU_3%_ gradually decreased from 25% to 19% (34%–27% when removing SSO_abs_) from samples from the shallowest HAUSGARTEN station HG-I to station HG-V (1,821 m total depth difference; Table S6). The same trend was observed for bacterial community structure (similarities in the relative abundance of OTU_3%_) with a gradual increase in dissimilarities of community structure with increasing water depth differences (Figure 4C). For the latitudinal transect, no significant correlation of community composition or structure with spatial distance was found (Figure 4D, Table S6).

**Figure 5:**
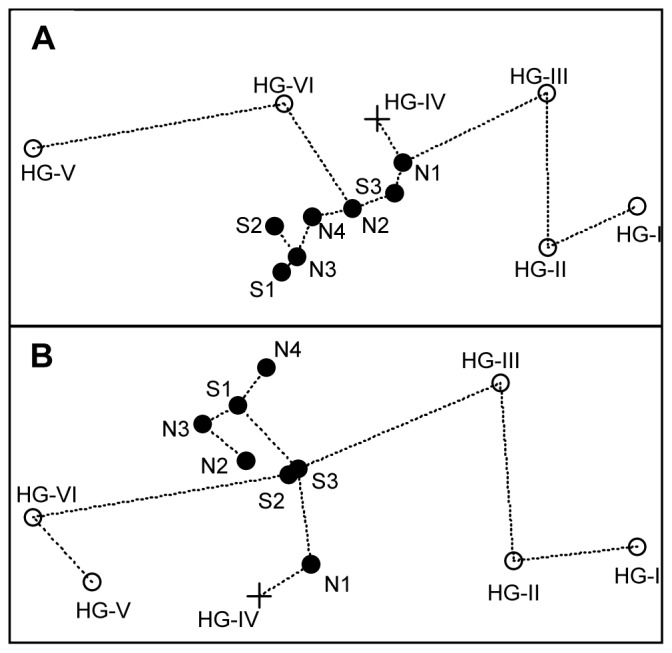
Non-metric multidimensional scaling (NMDS) plot of community data. MPTS (A) and ARISA (B) data based on Bray-Curtis dissimilarity matrices. Open circles indicate stations from the bathymetric transect, filled circles indicate stations from the latitudinal transect and the crosses indicate the central station. Dotted lines show a minimum spanning tree connecting nearest neighbours. Stress values: 0.05 for A and 0.06 for B.

In a non-metric multidimensional scaling plot (NMDS), visualizing dissimilarities of bacterial communities between samples, those from the bathymetric transect were located further apart from one another and had significantly higher community dispersion as those from the latitudinal transect (Figure 5). The latter samples grouped together and were significantly less dispersed (mean distances to their centroid of 0.21, as compared to 0.27 for samples from the bathymetric transect; 0.18 and 0.24, respectively, when removing SSO_abs_), as assessed by ANOVA of the distances to group centroids [41] (p = 0.003, p = 0.002 when removing SSO_abs_). These findings indicate that samples taken within a water depth zone were more similar to each other than across the zones. Grouping of the communities indicated higher similarities within the depth zones ∼1000–2000 m and>∼2500 m, which was previously also found for meiofauna taxa densities [13]. Strong bathymetric gradients, but without this clear zonation, were found for macro- and megafauna in Arctic deep-sea sediments (e.g. [55]).

### Spatial and Environmental Effects on Community Structure

We determined which environmental variables could explain some of the variation in bacterial community structure. In these analyses, bacterial community structure refers to the relative abundance of OTU_3%_ including singletons (analyses without SSO_abs_ led to the same conclusions; Table 1). Spatial variables consisted of longitude, latitude, spatial distance and water depth. Energy availability in the sediments in form of phytodetritus input from surface waters was estimated by measuring pigment concentrations (CPE). Porosity refers to the sediment water content. Protein and phospholipid concentrations were used to estimate total organic detritus and living microbial biomass, respectively. These environmental parameters have previously been shown to be related to differences in bacterial abundance, biomass and enzyme activities in Fram Strait (e.g. [22], [27]), and were hence chosen as proxies to represent some of the complex factors that may impact the variation in community structure at the LTER site HAUSGARTEN.

**Table 1:** Community response to spatial and environmental factors.

		OTU_3%_						OTU_ARISA_	
		All		SSO_abs_ removed		SSO_rel_ only			
		r^a^	R^2^ adj^b^.	r	R^2^ adj.	r	R^2^ adj.	r	R^2^ adj.
Spatial distance		∼	∼	∼	∼	∼	∼	∼	∼
Latitude		∼	∼	∼	∼	∼	∼	∼	∼
Longitude		0.38*	0.03*	0.42*	0.05*	∼	0.02*	0.47**	0.09*
Water depth		0.70**	0.07*** (0.05**)	0.71***	0.09*** (0.07**)	0.68**	0.06*** (0.04**)	0.83***	0.22*** (0.14**)
Phospholipids		0.31*	∼	0.36*	∼	0.49**	∼	0.45**	∼
CPE		0.38*	0.03* (∼)	0.36*	0.04*(∼)	0.33*	0.02* (∼)	0.38**	0.12** (∼)
covariation			(0.02)		(0.03)		(0.02)		(0.08)

OTU_3%_: Clustered sequences from MPTS at 97% sequence identity; OTU_ARISA_: OTU derived from ARISA fingerprinting; SSO_abs_: OTU_3%_ with only one sequence in the whole dataset (absolute singletons); SSO_rel_: OTU_3%_ with only one sequence in at least one sample but more than one sequence in the whole dataset (relative singletons). ^a^The significance of Spearman’s correlation coefficients (r) between relative OTU abundance tables and environmental parameter was determined by Mantel tests. ^b^Redundancy analysis (RDA) and partial RDA (pRDA; in brackets; to evaluate factor effect while taking the effects of other parameters into account) were used to determine the amount of variation (R^2^ adjusted) in the community data in a variation partitioning approach. For pRDA, the used parameters were water depth and CPE concentrations. Note that covariation effects cannot be tested for significance in the variation partitioning context (e.g. [71]). Significance levels are indicated as ***: p≤0.001, **: p≤0.01, *: p≤0.05, ∼: not significant, p>0.05.

Dissimilarities in bacterial community structure significantly increased with increasing differences in water depth (r = 0.70, p = 0.002) and longitude (r = 0.38, p = 0.017); Table 1), but not with latitude or spatial distance (p = 0.971 and p = 0.342, respectively). Water depth differences and bacterial community dissimilarity followed a continuous linear relationship within the investigated range of 1,284–3,535 m water depth (Figure 4). Redundancy analyses (RDA) revealed that water depth and longitude significantly explained 7% and 3% of variation in the OTU_3%_ dataset, respectively. Water depth was shown to correlate with bulk enzymatic activity, bacterial abundance and bacterial viability [13], [31]. A number of environmental factors vary with water depth and may include additional controlling factors, e.g. food quality or presence of larger organisms (e.g. nematodes [56]). In addition, adaptation to pressure differences might influence the bacterial community structure (e.g. [57]).

Particle flux of organic matter to the deep sea generally decreases with increasing water depth (e.g. [58], [59]). We observed that differences in CPE concentrations correlated positively with changes in bacterial community structure: stations with high differences in CPE concentrations showed more dissimilar community structures (r = 0.38, p = 0.019; Table 1, Figure 4) than those with similar CPE concentrations. A significant amount of 3% of the variation in bacterial community structure was explained by CPE concentrations (Table 1). Of course, CPE is just one proxy for phytodetritus input and does not necessarily reflect the complexity of food quantity and quality.

Although we did not find a significant correlation between water depth and CPE concentrations (p = 0.112; Table S5), they covaried and explained together with porosity 2% of the variation in community structure. Pure fractions of CPE concentrations (when the effect of covariation with water depth was removed) did not significantly explain variation in the community structure while pure fractions of water depth (when the effect of covariation with CPE was removed) still specifically explained 5% of the community variation. Porosity, proteins and phospholipids did not significantly explain variation in bacterial community structure (p = 0.313, p = 0.845 and p = 0.149, respectively) although differences in phospholipid concentrations significantly correlated with dissimilarities in community structure (r = 0.31, p = 0.04; Table 1). At the Siberian continental margin a relationship of bacterial community structure and phaeopigment concentration was found and a pure effect of phaeopigment concentrations (when the effect of water depth, spatial distance and protein concentrations was removed) could explain 5% of variation in community structure [11]. The reason why we did not find such a relationship could be explained by the smaller water depth range of this study (1284–3535 m water depth here, versus 37–3,427 m water depth at the Siberian continental margin), and the higher supply with phytodetritus at HAUSGARTEN.

Finally, we also tested the effect of grouping OTU_3%_ at coarser taxonomic resolution. In this case, community structure at every taxonomic level significantly correlated with differences in water depth and a high percentage (12% to 24%) of variation in community structure could be significantly explained (Table S7). This means that although most of the phyla and classes were common to all stations, their members significantly varied in relative abundances between different water depths. In contrast, no significant relationship between bacterial community structures at different taxonomic levels with CPE concentrations was found.

### Response of Individual Bacterial Taxa

Previous studies have shown that the abundance of Arctic deep-sea fauna either linearly decreased with decreasing water depth and food availability or peaked at intermediate water depth and thus phytodetritus input [60]. Therefore we used both linear and quadratic regression to test how individual bacterial taxa correspond to changes in water depth and CPE concentrations. Out of the 40 phyla identified in the dataset, 11 showed significant positive or negative relationships with increasing water depth (Table S8). Significant negative linear relationships with water depth were found for Verrucomicrobia and Planctomycetes, two related taxa which are ubiquitously found in soil and marine sediments, e.g. [61], [62], [63]. Their relevant contribution to benthic bacterial diversity was already reported from sediments in the Pacific sector of the Arctic Ocean [9], the Siberian margin [11] and coastal sites of Fram Strait [64], [65], yet no relationship with water depth had been detected. A positive quadratic relationship with water depth (minimum relative abundance at intermediate water depth) was found for Deferribacteres, which were previously found in coastal and deep-sea sediments [2], [40] and were reported from sediments from the Laptev Sea [11]. The phylum Actinobacteria showed a negative linear relationship with CPE concentrations, while Planctomycetes and Verrucomicrobia showed a positive linear relationship (Table S8). Verrucomicrobia was previously found to be also positively correlated to pigment concentrations in samples from the Siberian continental margin [11].

### Rare Biosphere

The rare bacterial biosphere was shown to make up a high fraction of bacterial community diversity in deep-sea sediments (e.g. [2]). Members of the rare biosphere include types which may vary in space and time and may become abundant when favourable conditions are present [66]. Here we looked at a subset of the rare biosphere including only those OTU_3%_ occurring with exactly one sequence in at least one sample but with more than one sequence in the whole dataset (“relative singletons“, SSO_rel_, [40]). This group of rare bacterial types comprised 31% of all OTU_3%_ (25% of all sequences), and on average 38±8% per sample. Interestingly, it showed similar responses to water depth changes and CPE concentrations as the whole community: water depth differences were highly correlated with differences in community structure and explained 6% of the variation in the SSO_rel_ community data, CPE concentrations correlated significantly with differences in community structure and explained 2% of the variation in the community (Table 1). When removing effects of covariation between water depth and CPE, the pure fraction of water depth still explained 4% of the variation in the SSO_rel_ community data, but pure fractions of CPE concentrations did not significantly explain any variation in the SSO_rel_ community data. This shows that the rare bacterial biosphere does vary with water depth, partly independent of phytodetritus concentrations. Likewise, differences in rare bacterial community structure with different water masses were found in the water column of the Arctic Ocean [67] and an effect of pigment concentrations on a part of low abundant bacterial types were reported from Arctic sediments [11].

Not only abundant types of bacteria but also rare members of the biosphere were found to be important for microbial processes (e.g. cellulose and chitin degradation [68]) and specific biogeochemical processes (e.g. sulphate reduction [69]). In Arctic sediments, high bacterial diversity was related to higher enzymatic activity and higher rates of organic matter degradation than in less diverse communities [65], and bacterial community patterns explained variations in enzyme activity [11]. Rare members of the biosphere might change in abundance with the varying availability of certain substrates (see [66] and references therein). Especially in variable environments such as the Arctic deep sea with a varying seasonal input of “fresh” phytodetritus, a high bacterial diversity and a complex community structure may be essential to react to environmental changes and for the functioning of the ecosystem [65], [68].

## Conclusions

We found a spatially highly diverse bacterial community in surface sediments of the Long-Term Ecological Research site HAUSGARTEN (Eastern Fram Strait). With 13 sampling stations over an area of about 3,385 km^2^ we assessed most of the estimated regional richness and found strong water depth related patterns of community structure along the bathymetric transect (54 km distance, 1,284–3,535 m water depth). Along the 120-km long latitudinal transect, no increasing bacterial community dissimilarity with increasing spatial distance could be observed. Nevertheless, a turnover of on average 79% OTU_3%_ (still 68% when absolute singletons were removed) was detected between any two samples taken within a distance of on average 13 km. Pigment concentrations as a proxy for energy supply in the form of phytodetritus sedimentation influenced bacterial community richness and structure, but no strong energy-diversity relationship was found within the investigated range. We identified indicator taxa that showed significant changes in relative sequence abundance with changes in water depth or pigment concentrations. This study demonstrates the complexity of bacterial community structure in deep-sea sediments and the necessity to investigate the regional biodiversity of deep-sea life not only at one single spot, but over scales of 1–100 km and different water depth zones, in order to better evaluate community responses related to environmental variations.

## Supporting Information

Figure S1 **OTU accumulation curves.**
(DOC)

Table S1 **List of samples taken during the Polarstern cruise ARK-XXIV/2 in 2009 and measured environmental parameters.**
(DOC)

Table S2 **Comparison of dataset structure based on ARISA and MPTS using Spearman correlation and Procrustes tests.**
(DOC)

Table S3 **Observed and estimated richness of OTU or taxa at different taxonomic levels and shared OTU or taxa between all stations.**
(DOC)

Table S4 **Observed and estimated richness of ARISA and MPTS data per station and in the total dataset.**
(DOC)

Table S5 **Spearman’s correlation matrix of alpha diversity measures, water depth and pigment concentrations (CPE).**
(DOC)

Table S6 **Percentages of pairwise shared, lost and gained OTU_3%_(A), OTU_3%_ without SSO_abs_ (B) and OTU_ARISA_ (C).**
(DOC)

Table S7 **Community response to water depth at different taxonomic levels.**
(DOC)

Table S8 **Linear and quadratic regression of phyla and classes in the OTU_3%_ dataset.**
(DOC)

Text S1 **Comparison of ARISA and MPTS and Richness of OTU.**
(DOC)
