# Supplementary material for: Biogeography of Deep-Sea Benthic Bacteria at Regional Scale (LTER HAUSGARTEN, Fram Strait, Arctic)
Source: PLoS One. 2013 Sep 2;8(9):e72779. doi: 10.1371/journal.pone.0072779 (PMC3759371; doi:10.1371/journal.pone.0072779)
Supplement: Figure S1 — OTU accumulation curves. (DOC) [file pone.0072779.s001.doc]

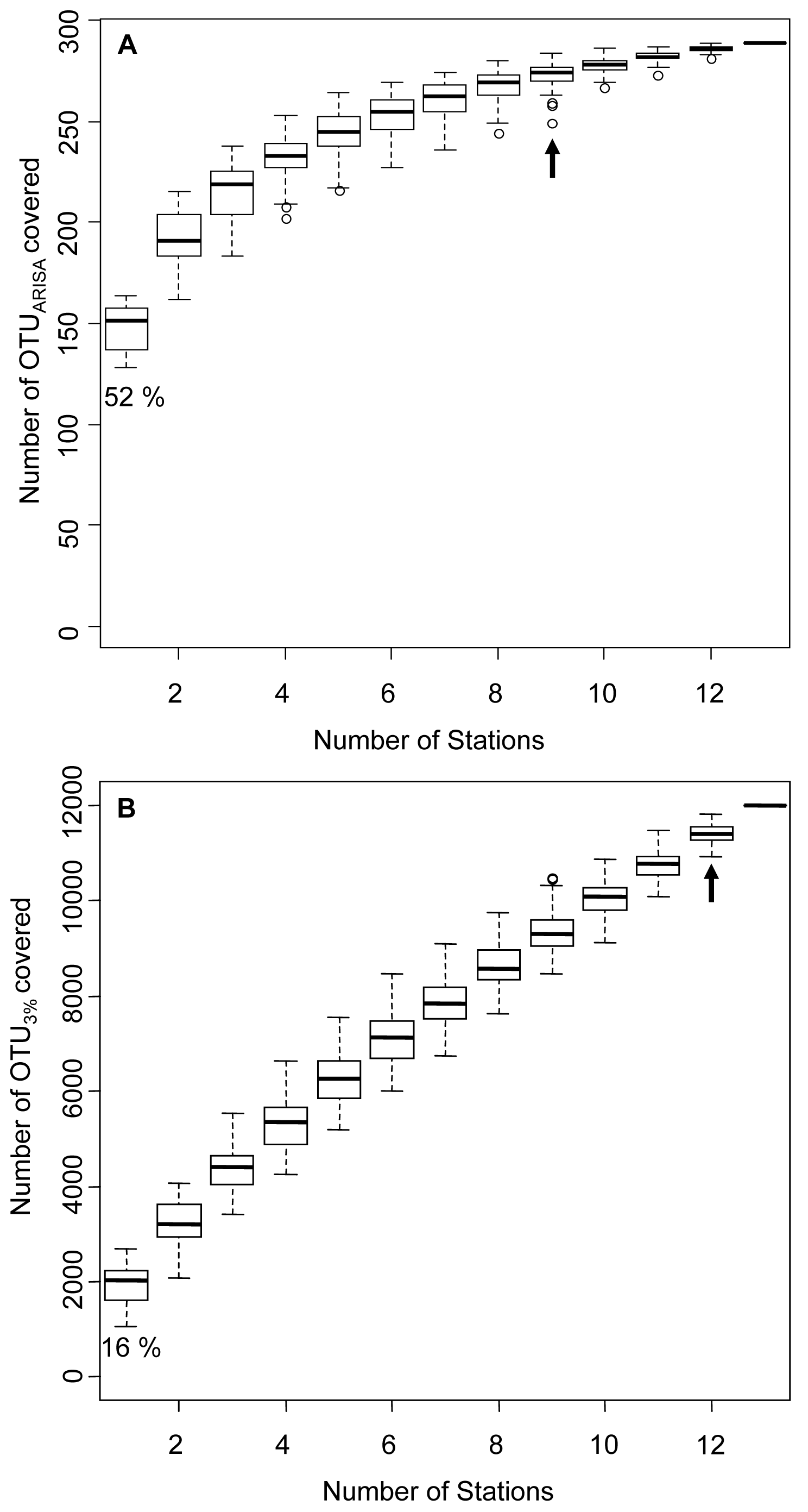


Figure S1. OTU accumulation curves. (A) based on ARISA data, (B) based on 454 MPTS data. The percentages indicated for n=1 station correspond to how much diversity would be recovered on average by randomly sampling only one station. The arrows indicate the number of stations needed to recover 95% of observed OTU.
