# Supplementary material for: Biogeography of Deep-Sea Benthic Bacteria at Regional Scale (LTER HAUSGARTEN, Fram Strait, Arctic)
Source: PLoS One. 2013 Sep 2;8(9):e72779. doi: 10.1371/journal.pone.0072779 (PMC3759371; doi:10.1371/journal.pone.0072779)
Supplement: Table S1 — List of samples taken during the Polarstern cruise ARK-XXIV/2 in 2009 and measured environmental parameters. (DOC) [file pone.0072779.s002.doc]

Table S1. List of samples taken during the Polarstern cruise ARK-XXIV/2 in 2009 and measured environmental parameters.

|  | Station | Water depth (m) | Latitude [N] | Longitude [E] | Porosity [% vol] | CPE* [µg cm^-3^] | Phospholipids [nmol ml^-1^] | Proteins [mg cm^-3^] | Event label | Date (2009) | Pangaea  Reference |
| --- | --- | --- | --- | --- | --- | --- | --- | --- | --- | --- | --- |
| Bathymetric transect | HG-I | 1284 | 79° 8' 2" | 6° 5' 46" | 72 ± 2 | 44 ± 5 | 13 ± 1 | 1.3 ± 0.2 | PS74/109-2 | 13 July | [1] |
|  | HG-II | 1547 | 79° 7' 48" | 4° 54' 7" | 63 ± 1 | 35 ± 8 | 6 ± 2 | 1.0 ± 0.1 | PS74/108-2 | 12 July | [2] |
|  | HG-III | 1895 | 79° 6' 29" | 4° 35' 56" | 55 ± 5 | 34 ± 7 | 13 ± 3 | 0.7 ± 0 | PS74/107-2 | 12 July | [3] |
|  | HG-IV (central st.) | 2464 | 79° 3' 50" | 4° 10' 55" | 55 ± 3 | 19 ± 3 | 9 ± 1 | 0.7 ± 0.1 | PS74/121-1 | 16 July | [4] |
|  | HG-V | 3105 | 79° 3' 47" | 3° 39' 32" | 58 ± 1 | 29 ± 8 | 17 ± 6 | 0.8 ± 0.1 | PS74/113-2 | 14 July | [5] |
|  | HG-VI | 3535 | 79° 3' 25" | 3° 34' 16" | 54 ± 6 | 21’ | 16 ± 2 | 0.4 ± 0.1 | PS74/106-3 | 12 July | [6] |
| Latitudinal transect | N1 | 2401 | 79° 16' 59" | 4° 19' 44" | 53 ± 2 | 19 ± 11 | 11 ± 6 | 1.1 ± 0.2 | PS74/120-2 | 16 July | [7] |
|  | N2 | 2545 | 79° 24' 36" | 4° 41' 24" | 66 ± 2 | 26 ± 4 | 15 ± 3 | 1.0 ± 0.6 | PS74/119-2 | 16 July | [8] |
|  | N3 | 2786 | 79° 36' 14" | 5° 10' 1" | 54 ± 4 | 31’ | 9 ± 1 | 3.0 ± 2.3 | PS74/118-2 | 16 July | [9] |
|  | N4 | 2802 | 79° 43' 1" | 4° 29' 10" | 57 ± 1 | 26 ± 7 | 11 ± 7 | 0.7 ± 0 | PS74/116-2 | 15 July | [10] |
|  | S1 | 2637 | 78° 55' 1" | 5° 0' 4" | 60 ± 1 | 24 ± 3 | 9 ± 3 | 0.5 ± 0.1 | PS74/127-2 | 17 July | [11] |
|  | S2 | 2473 | 78° 46' 48" | 5° 19' 37" | 66 ± 3 | 21 ± 2 | 11 ± 2 | 0.9 ± 0 | PS74/128-2 | 18 July | [12] |
|  | S3 | 2339 | 78° 36' 29" | 5° 4' 23" | 60 ± 2 | 30 ± 3 | 11 ± 2 | 1.2 ± 0 | PS74/129-3 | 18 July | [13] |

*CPE: Chloroplastic pigment equivalents used as proxy for phytodetritus input. ‘: no replicates were available.

References
