# Supplementary material for: Biogeography of Deep-Sea Benthic Bacteria at Regional Scale (LTER HAUSGARTEN, Fram Strait, Arctic)
Source: PLoS One. 2013 Sep 2;8(9):e72779. doi: 10.1371/journal.pone.0072779 (PMC3759371; doi:10.1371/journal.pone.0072779)
Supplement: Table S2 — Comparison of dataset structure based on ARISA and MPTS using Spearman correlation and Procrustes tests. (DOC) [file pone.0072779.s003.doc]

Table S2. Comparison of dataset structure based on ARISA and MPTS using Spearman correlation and Procrustes tests.

| Taxonomic level | Mantel test | Procrustes test |
| --- | --- | --- |
| Phylum | 0.33 * | 0.63 ** |
| Class | 0.35 * | 0.60 * |
| Order | 0.54 ** | ~ |
| Family | 0.57 ** | ~ |
| Genus | 0.63 *** | ~ |
| OTU_3%_ | 0.87*** | 0.83*** |

OTU_3%_: Clustered sequences from MPTS at 97% sequence identity. Significance: *: p < 0.05, **: p < 0.01, ***: p < 0.001, ~: not significant. Significance for Spearman correlation was determined by Mantel tests.
