# Supplementary material for: Biogeography of Deep-Sea Benthic Bacteria at Regional Scale (LTER HAUSGARTEN, Fram Strait, Arctic)
Source: PLoS One. 2013 Sep 2;8(9):e72779. doi: 10.1371/journal.pone.0072779 (PMC3759371; doi:10.1371/journal.pone.0072779)
Supplement: Table S3 — Observed and estimated richness of OTU or taxa at different taxonomic levels and shared OTU or taxa between all stations. (DOC) [file pone.0072779.s004.doc]

**Table S3.** **Observed and estimated richness of OTU or taxa at different taxonomic levels and shared OTU or taxa between all stations.**

|  | No. of observed taxa /OTU_ARISA_ | % OTU_3%_ annotated to taxonomic level | Chao1 richness estimator | % observed taxa of estimated taxa | No. of shared taxa/ OTU_ARISA_ between all stations | % of shared taxa/ OTU_ARISA_ between all stations |
| --- | --- | --- | --- | --- | --- | --- |
| Phylum | 41 | 99 | 42 | 99 | 27 | 66 |
| Class | 78 | 97 | 80 | 98 | 46 | 58 |
| Order | 136 | 89 | 139 | 98 | 73 | 51 |
| Family | 215 | 68 | 260 | 83 | 80 | 25 |
| Genus | 410 | 30 | 529 | 77 | 85 | 21 |
| OTU_3%_ | 12011 | 5 | 33778 | 36 | 217 | 2 |
| OTU_ARISA_ | 289 |  |  |  | 46 | 16 |

Abbreviations: OTU_ARISA_: Operational taxonomic unit as determined by binning ARISA peaks

with a window size 2; OTU_3%_: Clustered sequences from MPTS at 97% sequence identity.
