# Supplementary material for: Biogeography of Deep-Sea Benthic Bacteria at Regional Scale (LTER HAUSGARTEN, Fram Strait, Arctic)
Source: PLoS One. 2013 Sep 2;8(9):e72779. doi: 10.1371/journal.pone.0072779 (PMC3759371; doi:10.1371/journal.pone.0072779)
Supplement: Table S4 — Observed and estimated richness of ARISA and MPTS data per station and in the total dataset. (DOC) [file pone.0072779.s005.doc]

Table S4. Observed and estimated richness of ARISA and MPTS data per station and in the total dataset.

|  |  | No. of OTU_ARISA_ | No. of OTU_3%_ | No. of MPTS sequences | No. of SSO_abs_ | No. of SSO_rel_ | Chao1 richness estimates of MPTS |
| --- | --- | --- | --- | --- | --- | --- | --- |
| Bathymetric transect | HG-I | 133 | 1740 | 7382 | 423 | 657 | 2793 |
|  | HG-II | 150 | 1063 | 3716 | 179 | 516 | 2619 |
|  | HG-III | 153 | 2116 | 7408 | 703 | 785 | 4485 |
|  | HG-IV (central st.) | 140 | 1444 | 5793 | 343 | 627 | 2972 |
|  | HG-V | 137 | 1606 | 10993 | 384 | 570 | 2082 |
|  | HG-VI | 154 | 2236 | 14174 | 533 | 735 | 2482 |
| Latitudinal transect | N4 | 156 | 2351 | 12166 | 739 | 863 | 3411 |
|  | N3 | 158 | 1961 | 11020 | 572 | 718 | 3097 |
|  | N2 | 164 | 2017 | 11534 | 475 | 800 | 2542 |
|  | N1 | 128 | 2572 | 14943 | 643 | 880 | 2904 |
|  | S1 | 157 | 2397 | 13192 | 831 | 790 | 3511 |
|  | S2 | 163 | 2671 | 13264 | 1036 | 850 | 3729 |
|  | S3 | 159 | 2196 | 11490 | 569 | 820 | 2502 |
|  | Total* | 289 | 12011 | 137075 | 7430 | 3705 | 33778 |

Abbreviations: OTU_ARISA_: Operational taxonomic unit as determined by binning ARISA peaks with a window size 2; OTU_3%_: Clustered sequences from MPTS at 97% sequence identity; SSO_abs_ (absolute singletons): OTU_3%_ with only one sequence in the whole dataset. SSO_rel_ (relative singletons): OTU_3%_ with only one sequence in a given sample but more than one sequence in the whole dataset; Chao1 richness estimates per station were calculated on normalized data based on the least abundant one (HG-II, 3,716 sequences). *: Total numbers in the whole dataset.
