# Supplementary material for: Biogeography of Deep-Sea Benthic Bacteria at Regional Scale (LTER HAUSGARTEN, Fram Strait, Arctic)
Source: PLoS One. 2013 Sep 2;8(9):e72779. doi: 10.1371/journal.pone.0072779 (PMC3759371; doi:10.1371/journal.pone.0072779)
Supplement: Table S5 — Spearman’s correlation matrix of alpha diversity measures, water depth and pigment concentrations (CPE). (DOC) [file pone.0072779.s006.doc]

**Table S5. Spearman’s correlation matrix of alpha diversity measures, water depth and pigment concentrations (CPE).**

|  | Water depth | CPE | OTU_3%_ | SSO_abs_ | SSO_rel_ | Chao1 richness estimator | OTU_3%_ without SSO_abs_ | OTU_ARISA_ | reads |
| --- | --- | --- | --- | --- | --- | --- | --- | --- | --- |
| Water depth |  | 0.112 | 0.415 | 0.494 | 0.517 | 0.541 | 0.364 | 0.364 | 0.082 |
| CPE | -0.46 |  | 0.058 | 0.344 | 0.078 | 0.845 | **0.044** | 1.000 | ***0.012*** |
| OTU_3%_ | 0.25 | -0.54 |  | ***0.000*** | ***0.000*** | 0.168 | ***0.000*** | 0.263 | ***0.000*** |
| SSO_abs_ | 0.21 | -0.29 | ***0.89*** |  | ***0.000*** | ***0.008*** | ***0.010*** | 0.144 | ***0.011*** |
| SSO_rel_ | 0.20 | -0.51 | ***1.00*** | ***0.90*** |  | 0.162 | ***0.000*** | 0.231 | ***0.000*** |
| Chao1 richness estimator | -0.20 | -0.06 | 0.41 | ***0.70*** | 0.41 |  | 0.762 | 0.566 | 0.817 |
| OTU_3%_ without SSO_abs_ | 0.28 | **-0.57** | ***0.92*** | ***0.70*** | ***0.91*** | 0.09 |  | 0.334 | ***0.000*** |
| OTU_ARISA_ | 0.28 | 0.00 | 0.34 | 0.43 | 0.36 | 0.18 | 0.29 |  | 0.334 |
| reads | 0.50 | ***-0.67*** | ***0.90*** | ***0.68*** | ***0.87*** | 0.07 | ***0.93*** | 0.29 |  |

Upper matrix triangle indicates p-values; lower matrix triangle indicates the Spearman’s correlation value. Bold font indicates significant correlations, italic font indicates still significance after correction for multiple comparisons using the false discovery rate. Longitude, latitude, porosity, proteins and phospholipids did not show significant correlations and are therefore not shown.
