# Supplementary material for: Biogeography of Deep-Sea Benthic Bacteria at Regional Scale (LTER HAUSGARTEN, Fram Strait, Arctic)
Source: PLoS One. 2013 Sep 2;8(9):e72779. doi: 10.1371/journal.pone.0072779 (PMC3759371; doi:10.1371/journal.pone.0072779)
Supplement: Table S6 — Percentages of pairwise shared, lost and gained OTU3%(A), OTU3% without SSOabs (B) and OTUARISA (C). (DOC) [file pone.0072779.s007.doc]

**Table S6**. **Percentages of pairwise shared, lost and gained OTU_3%_(A), OTU_3%_ without SSO_abs_ (B) and OTU_ARISA_ (C).**

|  | **A** | HG-I | HG-II | HG-III | HG-IV | HG-V | HG-VI | N4 | N3 | N2 | N1 | S1 | S2 | S3 |
| --- | --- | --- | --- | --- | --- | --- | --- | --- | --- | --- | --- | --- | --- | --- |
| Bathymetric transect | HG-I |  | 53 / 23 | 33 / 45 | 45 / 34 | 43 / 38 | 32 / 47 | 31 / 49 | 36 / 43 | 34 / 43 | 27 / 50 | 31 / 50 | 28 / 53 | 32 / 46 |
|  | HG-II | 25 |  | 20 / 60 | 30 / 49 | 29 / 53 | 20 / 62 | 19 / 63 | 22 / 58 | 20 / 58 | 15 / 65 | 19 / 64 | 17 / 67 | 19 / 61 |
|  | HG-III | 22 | 21 |  | 52 / 29 | 49 / 33 | 38 / 41 | 37 / 43 | 42 / 38 | 41 / 38 | 33 / 44 | 37 / 45 | 34 / 48 | 38 / 40 |
|  | HG-IV | 21 | 21 | 19 |  | 37 / 43 | 26 / 52 | 25 / 54 | 30 / 48 | 28 / 49 | 21 / 56 | 25 / 55 | 23 / 59 | 26 / 51 |
|  | HG-V | 19 | 19 | 18 | 21 |  | 28 / 49 | 29 / 51 | 34 / 46 | 32 / 46 | 26 / 54 | 29 / 52 | 26 / 55 | 31 / 49 |
|  | HG-VI | 21 | 19 | 20 | 22 | 23 |  | 37 / 40 | 43 / 35 | 41 / 35 | 33 / 42 | 37 / 41 | 35 / 45 | 39 / 38 |
| Latitudinal transect | N4 | 21 | 18 | 20 | 21 | 20 | 22 |  | 44 / 33 | 43 / 34 | 35 / 41 | 39 / 40 | 36 / 44 | 41 / 37 |
|  | N3 | 21 | 20 | 20 | 22 | 21 | 22 | 22 |  | 38 / 39 | 30 / 46 | 33 / 45 | 31 / 49 | 35 / 42 |
|  | N2 | 22 | 22 | 21 | 23 | 22 | 24 | 23 | 23 |  | 29 / 44 | 34 / 44 | 31 / 48 | 35 / 40 |
|  | N1 | 23 | 20 | 23 | 23 | 21 | 24 | 24 | 24 | 26 |  | 41 / 37 | 38 / 40 | 42 / 32 |
|  | S1 | 19 | 18 | 18 | 20 | 19 | 21 | 21 | 22 | 22 | 22 |  | 37 / 44 | 42 / 36 |
|  | S2 | 19 | 16 | 18 | 18 | 19 | 20 | 20 | 20 | 21 | 22 | 19 |  | 45 / 34 |
|  | S3 | 22 | 21 | 22 | 23 | 20 | 23 | 23 | 23 | 25 | 26 | 22 | 21 |  |

|  | b)b**B** | HG-I | HG-II | HG-III | HG-IV | HG-V | HG-VI | N4 | N3 | N2 | N1 | S1 | S2 | S3 |
| --- | --- | --- | --- | --- | --- | --- | --- | --- | --- | --- | --- | --- | --- | --- |
| Bathymetric transect | HG-I |  | 46 / 20 | 30 / 35 | 41 / 29 | 39 / 34 | 27 / 44 | 28 / 44 | 33 / 36 | 29 / 39 | 21 / 36 | 30 / 41 | 28 / 41 | 27 / 41 |
|  | HG-II | 34 |  | 20 / 50 | 29 / 43 | 28 / 48 | 18 / 57 | 18 / 55 | 21 / 50 | 18 / 53 | 13 / 60 | 19 / 54 | 18 / 56 | 17 / 55 |
|  | HG-III | 35 | 31 |  | 43 / 27 | 41 / 31 | 28 / 41 | 29 / 38 | 35 / 33 | 31 / 37 | 22 / 43 | 31 / 38 | 30 / 39 | 28 / 38 |
|  | HG-IV | 30 | 28 | 30 |  | 32 / 39 | 21 / 49 | 22 / 46 | 26 / 41 | 23 / 45 | 15 / 52 | 23 / 46 | 22 / 48 | 21 / 46 |
|  | HG-V | 27 | 25 | 28 | 29 |  | 23 / 45 | 26 / 44 | 30 / 39 | 27 / 42 | 21 / 50 | 27 / 43 | 25 / 44 | 27 / 45 |
|  | HG-VI | 29 | 25 | 31 | 31 | 33 |  | 35 / 31 | 40 / 27 | 36 / 30 | 28 / 37 | 36 / 31 | 35 / 32 | 35 / 32 |
| Latitudinal transect | N4 | 31 | 27 | 32 | 32 | 30 | 34 |  | 37 / 27 | 34 / 31 | 26 / 38 | 33 / 32 | 32 / 33 | 32 / 33 |
|  | N3 | 31 | 29 | 32 | 33 | 31 | 33 | 35 |  | 29 / 36 | 21 / 43 | 28 / 36 | 28 / 39 | 27 / 38 |
|  | N2 | 32 | 30 | 32 | 33 | 30 | 34 | 35 | 34 |  | 23 / 39 | 33 / 34 | 31 / 35 | 30 / 34 |
|  | N1 | 33 | 28 | 36 | 33 | 30 | 35 | 37 | 36 | 38 |  | 40 / 26 | 38 / 27 | 37 / 25 |
|  | S1 | 29 | 27 | 31 | 31 | 29 | 33 | 35 | 35 | 34 | 35 |  | 32 / 35 | 31 / 34 |
|  | S2 | 31 | 26 | 31 | 30 | 31 | 33 | 35 | 34 | 34 | 36 | 34 |  | 33 / 32 |
|  | S3 | 32 | 29 | 34 | 33 | 28 | 33 | 35 | 35 | 36 | 38 | 35 | 35 |  |

|  | b)b**C** | HG-I | HG-II | HG-III | HG-IV | HG-V | HG-VI | N4 | N3 | N2 | N1 | S1 | S2 | S3 |
| --- | --- | --- | --- | --- | --- | --- | --- | --- | --- | --- | --- | --- | --- | --- |
| Bathymetric transect | HG-I |  | 12 / 22 | 17 / 28 | 28 / 31 | 29 / 31 | 25 / 35 | 23 / 34 | 24 / 36 | 21 / 36 | 29 / 27 | 25 / 36 | 20 / 35 | 21 / 34 |
|  | HG-II | 66 |  | 19 / 21 | 29 / 23 | 31 / 25 | 27 / 29 | 25 / 28 | 25 / 29 | 21 / 28 | 31 / 19 | 24 / 27 | 20 / 26 | 21 / 25 |
|  | HG-III | 55 | 59 |  | 30 / 24 | 33 / 25 | 28 / 28 | 24 / 25 | 27 / 29 | 20 / 26 | 31 / 17 | 25 / 26 | 22 / 27 | 20 / 23 |
|  | HG-IV | 41 | 48 | 46 |  | 23 / 21 | 22 / 29 | 17 / 26 | 14 / 23 | 11 / 24 | 21 / 14 | 14 / 23 | 13 / 26 | 10 / 20 |
|  | HG-V | 39 | 44 | 42 | 56 |  | 13 / 23 | 16 / 26 | 17 / 28 | 14 / 28 | 24 / 19 | 17 / 28 | 15 / 29 | 16 / 28 |
|  | HG-VI | 40 | 45 | 44 | 49 | 64 |  | 21 / 22 | 22 / 24 | 20 / 25 | 30 / 16 | 22 / 24 | 20 / 25 | 21 / 23 |
| Latitudinal transect | N4 | 43 | 47 | 51 | 57 | 58 | 57 |  | 16 / 17 | 14 / 18 | 27 / 11 | 15 / 16 | 15 / 18 | 13 / 15 |
|  | N3 | 39 | 47 | 45 | 63 | 55 | 54 | 66 |  | 11 / 15 | 28 / 11 | 15 / 14 | 14 / 17 | 17 / 17 |
|  | N2 | 43 | 52 | 54 | 65 | 58 | 55 | 68 | 74 |  | 27 / 7 | 14 / 10 | 14 / 14 | 14 / 11 |
|  | N1 | 44 | 49 | 52 | 65 | 57 | 54 | 61 | 61 | 66 |  | 9 / 26 | 7 / 27 | 6 / 25 |
|  | S1 | 39 | 49 | 49 | 63 | 56 | 54 | 69 | 71 | 76 | 65 |  | 12 / 16 | 12 / 13 |
|  | S2 | 45 | 54 | 50 | 61 | 56 | 55 | 67 | 69 | 72 | 65 | 72 |  | 13 / 11 |
|  | S3 | 46 | 54 | 57 | 70 | 56 | 56 | 72 | 66 | 75 | 69 | 75 | 76 |  |

Lower-matrix triangle: Pairwise shared OTU; upper-matrix triangle: percentages of unique OTU in the station on the row and column respectively.

For example, 25 % of OTU_3%_ (a) are shared between stations HG-I and HG-II, 53 % are unique to station HG-I and 23 % are unique to station HG-II.
