# Supplementary material for: Biogeography of Deep-Sea Benthic Bacteria at Regional Scale (LTER HAUSGARTEN, Fram Strait, Arctic)
Source: PLoS One. 2013 Sep 2;8(9):e72779. doi: 10.1371/journal.pone.0072779 (PMC3759371; doi:10.1371/journal.pone.0072779)
Supplement: Table S7 — Community response to water depth at different taxonomic levels. (DOC) [file pone.0072779.s008.doc]

Table S7. Community response to water depth at different taxonomic levels.

|  | OTU_3%_ | | | | | | | |
| --- | --- | --- | --- | --- | --- | --- | --- | --- |
|  | | All | | SSO_rel_ only | | SSO_abs_ removed | |  |
|  | | r^b^ | R^2^ adj.^b^ | r | R^2^ adj. | r | R^2^ ad.j |  |
| Phylum | | 0.29* | 24** | ~ | 14** | 0.26* | 23** |  |
| Class | | 0.30* | 23** | 0.44** | 19** | 0.29* | 22** |  |
| Order | | 0.34* | 13* | 0.48** | 14** | 0.33* | 13* |  |
| Family | | 0.39* | 13* | 0.39** | 13** | 0.37* | 13* |  |
| Genus | | 0.52** | 12* | 0.57** | 13** | 0.47* | 14* |  |
| OTU_3%_ | | 0.70*** | 7** | 0.68*** | 6* | 0.71*** | 9*** |  |

OTU_3%_: Clustered sequences from MPTS at 97% sequence identity; SSO_rel_: OTU_3%_ with only one sequence in at least one sample but more than one sequence in the whole dataset. SSO_abs_: OTU_3%_ with only one sequence in the whole dataset (absolute singletons);^a^The significance of Spearman’s correlation coefficients (r) between relative OTU abundance tables and water depth was determined by Mantel tests. ^b^Redundancy analysis (RDA) was used to determine the amount of variation (R^2^ adjusted) in the community data that can be explained by water depth. Significance levels are indicated as *** p ≤ 0.001, ** p ≤ 0.01, * p ≤ 0.05, ~: not significant p > 0.05.
