# Supplementary material for: Biogeography of Deep-Sea Benthic Bacteria at Regional Scale (LTER HAUSGARTEN, Fram Strait, Arctic)
Source: PLoS One. 2013 Sep 2;8(9):e72779. doi: 10.1371/journal.pone.0072779 (PMC3759371; doi:10.1371/journal.pone.0072779)
Supplement: Table S8 — Linear and quadratic regression of phyla and classes in the OTU3% dataset. (DOC) [file pone.0072779.s009.doc]

**Table S8. Linear and quadratic regression of phyla and classes in the OTU_3%_** dataset.

|  |  |  |  |  |  |  |  |  |  |  |  |
| --- | --- | --- | --- | --- | --- | --- | --- | --- | --- | --- | --- |
|  |  |  | water depth | | | | CPE | | | |  |
|  |  |  | lm | | qm | | lm | | qm | |  |
|  |  | No. of seq. | R^2^ | sign | R^2^ | sign | R^2^ | sign | R^2^ | sign | # |
| phyla | Actinobacteria | 7988 |  |  |  |  | 0.38 | - |  |  | 13 |
|  | Acidobacteria | 6720 | 0.31 | - |  |  |  |  |  |  | 13 |
|  | Verrucomicrobia | 6505 | 0.64* | - |  |  | 0.30 | + |  |  | 13 |
|  | Planctomycetes | 5227 | 0.63* | - |  |  | 0.27 | + | 0.49 | + | 13 |
|  | Deferribacteres | 978 | 0.43 | + | 0.62 | + |  |  |  |  | 13 |
|  | Thermodesulfobacteria | 844 |  |  | 0.25 | - |  |  | 0.65 | + | 13 |
|  | Lentisphaerae | 647 | 0.34 | - |  |  |  |  |  |  | 13 |
|  | Candidate division  OP3 | 354 |  |  |  |  |  |  | 0.35 | + | 13 |
|  | BD1-5 | 320 | 0.31 | + |  |  |  |  |  |  | 13 |
|  | Candidate division TM6 | 230 |  |  | 0.37 | - |  |  | 0.32 | + | 13 |
|  | Chlorobi | 208 |  |  |  |  |  |  | 0.27 | + | 13 |
|  | Candidate division TG-1 | 104 |  |  | 0.45 | + |  |  |  |  | 13 |
|  | NPL-UPA2 | 72 |  |  | 0.30 | - |  |  |  |  | 11 |
|  | Deinococcus-Thermus | 53 | 0.25 | - |  |  |  |  |  |  | 11 |
|  | GOUTA4 | 19 |  |  |  |  |  |  | 0.23 | - | 10 |
| class | Actinobacteria | 7988 |  |  |  |  | 0.38 | - |  |  | 13 |
|  | Acidobacteria | 3563 | 0.26 | - |  |  |  |  |  |  | 13 |
|  | Alphaproteobacteria | 6154 | 0.48 | - |  |  | 0.36 | + | 0.62 | + | 13 |
|  | Verrucomicrobiae | 4912 | 0.60* | - |  |  | 0.31 | + |  |  | 13 |
|  | Planctomycetacia | 2108 | 0.70* | - | 0.86 | + | 0.39 | + | 0.65 | + | 13 |
|  | RB25 | 2062 | 0.35 | - |  |  |  |  |  |  | 13 |
|  | Unclassified  Deferribacterales | 975 | 0.43 | + | 0.62 | + |  |  |  |  | 13 |
|  | JTB23 | 870 |  |  |  |  |  |  | 0.31 | + | 13 |
|  | Thermodesulfobacteria | 844 |  |  | 0.25 | - |  |  |  |  | 13 |
|  | OM190 | 670 |  |  |  |  |  |  |  |  | 13 |
|  | Opitutae | 657 | 0.48 | - |  |  |  |  |  |  | 13 |
|  | Lentisphaeria | 647 | 0.34 | - |  |  |  |  |  |  | 13 |
|  | TA18 | 264 |  |  |  |  |  |  |  |  | 13 |
|  | Chlorobia | 208 |  |  |  |  |  |  | 0.27 | + | 13 |
|  | OPB35 | 134 |  |  | 0.45 | + |  |  |  |  | 13 |
|  | KD4-96 | 106 | 0.38 | - |  |  |  |  |  |  | 13 |
|  | Candidatus Kuenenia | 42 | 0.53 | - |  |  | 0.35 | + |  |  | 13 |
|  | Thermales | 39 | 0.29 | - |  |  |  |  |  |  | 11 |
|  | Lineage I  Endomicrobia | 31 | 0.30 | + |  |  | 0.28 | - |  |  | 11 |
|  | Acidimethylosilex | 25 |  |  |  |  | 0.29 | - |  |  | 9 |
|  | GIF3 | 10 | 0.36 | + |  |  |  |  |  |  | 7 |

Only those phyla and classes which showed a significant relation are shown here (p < 0.05); * indicates still significance after correction for multiple comparisons using the false discovery rate. Linear model: - and + indicate decrease or increase with increasing water depth or CPE concentrations, respectively; for quadratic model: - and + indicate maximum or minimum relative abundance at intermediate water depth or CPE concentrations, respectively. # indicates the number of samples where a taxon was present.
