# Supplementary material for: Biogeography of Deep-Sea Benthic Bacteria at Regional Scale (LTER HAUSGARTEN, Fram Strait, Arctic)
Source: PLoS One. 2013 Sep 2;8(9):e72779. doi: 10.1371/journal.pone.0072779 (PMC3759371; doi:10.1371/journal.pone.0072779)
Supplement: Text S1 — Comparison of ARISA and MPTS and Richness of OTU. (DOC) [file pone.0072779.s010.doc]

**Text S1**

**Comparison of ARISA and MPTS**

Shifts in bacterial community structure were investigated using automated ribosomal intergenic spacer analysis (ARISA) and 454 massively parallel tag sequencing (MPTS) of the V4-V6 variable regions, which are both commonly used techniques to describe microbial communities over large spatial scales (e.g. [1,2]). The two techniques differ to some extent: ARISA targets the length variability of the 16-23S intergenic region, but is limited in the number of detectable operational taxonomic units (OTU), thus representing abundant types of bacteria, which limits the use of richness estimates [3]. Moreover, it does not provide phylogenetic information [4]. In contrast, MPTS offers an in-depth view on community composition (based on presence or absence of OTU) and structure (based on the relative abundance of OTU) ,e.g. [5,6]. At the resolution of family to genus, both community fingerprinting methods show highly congruent patterns (e.g. [7], Gobet et al. in preparation). Also in this study, we found consistent community patterns derived from both data types at different taxonomic resolution levels (Table S2). In this study, we mostly focused on results based on MPTS data, including some comparisons to the patterns detected by ARISA.

**Richness**

On average 2,028 ± 463 OTU_3%_ occurred per sample at each station. After removing pyrosequencing and PCR-related technical errors, on average 572 ± 225 OTU_3%_ per sample were absolute singletons (SSO_abs_; Table S4). This resulted in 7,430 SSO_abs_ (62% of all OTU_3%_, 5% of all denoised sequences) in the whole dataset. In total, 3,705 OTU_3%_ (31% of all OTU_3%_, 25% of all denoised sequences) were relative singletons (SSO_rel_) with on average 739 ± 116 SSO_rel_ per sample. Overall this indicates that a large fraction of the recovered bacterial diversity consisted of rare microbial types. Noticeably, total number of OTU_3%_, SSO_abs_ and SSO_rel_ were all correlated positively to each other (Table S5), indicating that more rare types (either absolute or with fluctuating sequence abundances) were discovered as observed richness increased.

References

1. Fuhrman JA, Steele JA, Hewson I, Schwalbach MS, Brown MV, et al. (2008) A latitudinal diversity gradient in planktonic marine bacteria. Proceedings of the National Academy of Sciences, USA 105: 7774-7778.

2. Zinger L, Amaral-Zettler LA, Fuhrman JA, Horner-Devine MC, Huse SM, et al. (2011) Global patterns of bacterial beta-diversity in seafloor and seawater ecosystems. PLoS ONE 6: e24570.

3. Bent SJ, Pierson JD, Forney LJ, Danovaro R, Luna GM, et al. (2007) Measuring species richness based on microbial community fingerprints: The emperor has no clothes. Applied and Environmental Microbiology 73: 2399-2401.

4. Zinger L, Gobet A, Pommier T (2012) Two decades of describing the unseen majority of aquatic microbial diversity. Molecular Ecology 21: 1878-1896.

5. Galand PE, Casamayor EO, Kirchman DL, Lovejoy C (2009) Ecology of the rare microbial biosphere of the Arctic Ocean. Proceedings of the National Academy of Sciences, USA 106: 22427-22432.

6. Sogin ML, Morrison HG, Huber JA, Welch DM, Huse SM, et al. (2006) Microbial diversity in the deep sea and the underexplored "rare biosphere". Proceedings of the National Academy of Sciences, USA 103: 12115-12120.

7. Bienhold C, Boetius A, Ramette A (2012) The energy-diversity relationship of complex bacterial communities in Arctic deep-sea sediments. ISME Journal 6: 724-732.
